# The complex translocation (9;14;14) involving *IGH* and *CEBPE* genes suggests a new subgroup in B-lineage acute lymphoblastic leukemia

**DOI:** 10.1590/S1415-475738420140368

**Published:** 2016

**Authors:** Rachid Zerrouki, Traki Benhassine, Mustapha Bensaada, Patricia Lauzon, Anissa Trabzi

**Affiliations:** 1 Laboratoire de Biologie Cellulaire et Moléculaire, Faculté des Sciences Biologiques, Université des Sciences et Technologies Houari Boumediene, Alger, Algeria; 2 Centre Pierre et Marie-Curie, Service d'Onco-Hématologie & Hôpital Mustapha Bacha, Alger, Algeria; 3 Clinique de Chirurgie et des Sciences de la Reproduction, Laboratoire de Cytogénétique, Constantine, Algeria; 4 Animal Health Unit, University of Calgary, Calgary, Alberta, Canada

**Keywords:** acute lymphoblastic leukemia, CEBPE, FISH, IGH, translocation

## Abstract

Many subtypes of acute lymphoblastic leukemia (ALL) are associated with specific chromosomal rearrangements. The complex translocation t(9;14;14), a variant of the translocation (14;14)(q11;q32), is a rare but recurrent chromosomal abnormality involving the immunoglobulin heavy-chain (*IGH*) and CCAAT enhancer-binding protein (*CEBPE*) genes in B-lineage ALL (B-ALL) and may represent a new B-ALL subgroup. We report here the case of a 5-year-old girl with B-ALL, positive for CD19, CD38 and HLA-DR. A direct technique and G-banding were used for chromosomal analysis and fluorescent*in situ* hybridization (FISH) with BAC probes was used to investigate a possible rearrangement of the *IGH* and*CEBPE* genes. The karyotype exhibit the chromosomal aberration 46,XX,del(9)(p21),t(14;14)(q11;q32). FISH with dual-color break-apart*IGH*-specific and *CEPBE*-specific bacterial artificial chromosome (BAC) probes showed a complex t(9;14;14) associated with a deletion of cyclin-dependent kinase inhibitor 2A (*CDKN2A*) and paired box gene 5 (*PAX5*) at 9p21-13 and duplication of the fusion gene *IGH-CEBPE*.

## Introduction

Acute lymphoblastic leukemia (ALL) is a malignant clonal proliferation of lymphoid progenitor cells, most commonly of the B-cell lineage (B-ALL). In pediatric populations, ALL accounts for 81% of childhood leukemias, with leukemia in general accounting for one third of cancers diagnosed in children up to 14 years of age (Howlader *et al.*, 2014;Woo *et al.*, 2014).

The translocation (14;14)(q11;q32) is a reciprocal translocation and a variant of inv(14)(q11q32). These rearrangements should not be confused with t(14;14)(q11;q32) and inv(14)(q11q32) seen in T-cell malignancies in ataxiatelangiectasia (AT) patients and non-AT patients (Bertness *et al.*, 1990; Matutes*et al.*, 1991; Minegishi *et al.*, 1991; Taylor *et al.*, 1996; Przybylski *et al.*, 2005; Graux *et al.*, 2006; Haider *et al.*, 2006). The former involves the T-cell receptor (TCR) loci *TCRA* at 14q11 and *TCL1* at 14q32. The same chromosomal rearrangements in B-ALL involve the *IGH*(14q32) and *CEBPE* (14q11) loci (Table 1).

**Table 1:** Clinical, hematological, immunophenotypic and genetic findings in T & B-ALL patients with t(14;14)(q11;q32) and inv(14)(q11q32). Modified fromHan *et al.*(2008).

Case	Age (yr)/sex	Karyotype	FISH Molecular analysis	Immunophenotype	BM blast cells (%)	WBC x l0^9^/L	References
1	12/M	43,XY,t(14;14)(q11;q32),-2,+3mar,-7,−13,−16, −17,−18,−19,-21,+ring(7 ?),+der(2)t(2;?)(p25;?), +der(17)t(11;17) (q13;pl2	TCR involvement	CD3+, CD7+, CD8+	NA	NA	Minegishi *et al.*(1991)
2	Adult/M	45,X,-Y,add(1)(q10),t(1;3)(p12;q25),+i(1)(q10), Add(7) (p22),−10,del(13)(q22q32),inv(14) (q11q32),Add(21)(p11),-22,+mar[27]/46,XY[2]	TCR involvement	CD2+, CD3+, CD4+, CD5+, CD25+	NA	NA	Haider *et al.*(2006)
3	5.6/F	45,XX,-7,t(14;14)(q11;q32)[14]/46,XX[2]	NA	B lineage	NA	38.7	Raimondi *et al.*(2003)
4	7/F	46,XX,t(14;14)(q11;q32))[13]/46,XX[l]	Breakpoints, were located telomeric, to the TCR and*IGH* loci	CD10+, CD19+, CD38+	85	171	Shiloh & Cohen (1978)
5	36/F	46,XX,del(6)(q32),t(14;14)(q11;q32)[20]	*IGH,* involvement	CD10+, CD19+, CD22+, CD38+	85	41.1	Liu***et*** *al.*(2004)
6	44/M	47,XY,t(14;14)(q11;q32),+mar[15]/46,XY[5]	*IGH,* involvement	CD9+, CD10+, CD19+, CD20+, CD22+, CD38+	92.5	73.6	Liu***et*** *al.*(2004)
7	45/M	45,XY,dup(5)(q14q21),-7,t(14;14)(q11;q32)[17]	*IGH* and *CEBPE,*involvement	CD10+, CD19+, CD34+, CD38+	NA	1	Akasaka *et al.*(2007)
8	39/F	47,XX,+4,t(14;14)(q11;q32)[20]	*IGH* and *CEBPE,*involvement	CD10+,CD19+,CD79a+	88.5	3.6	Han ***et al.*** (2008)
9	5/F	46,XX,del(9)(p21),t(9;14;14)(p12;q11;q32)[20]	*IGH* and *CEBPE*(*PAX5* and *CDKN2A,* deletion) involvement	CD10+, CD19+, CD22+, CD33+, CD34+, CD38+, CD45+, CD79b+, HLA-DR+	90	3.9	Present case

Abbreviations: BM, bone marrow; CCAAT, enhancer-binding protein; CD, cluster differentiation; CEBPE, CDKN2A, cyclin-dependent kinase inhibitor 2A; F, female; HLA-DR, human leucocyte antigen; IGH, immunoglobulin heavy chain; L, liter; M, male; NA, not available; PAX5, paired box gene 5; TCR, T-cell receptor; WBC, white blood cell; yr, year.

*CEBPE* is part of the five-member *CEBP* gene family: CEBPA (19q13), *CEBPB* (20q13), *CEBPD* (8q11),*CEBPE* (14q11) and *CEBPG* (19q13) (Lekstrom-Himes, 2001). In B-ALL patients with t(14;14)(q11;q32), *CEBPE* plays an oncogenic role in the pathogenesis of this leukemia (Truong *et al.*, 2003). Involvement of the *IGH* gene in this translocation was demonstrated by Liu*et al.* (2004) using a dual-color break-apart*IGH* probe (Abott/Vysis, USA). Akasaka *et al.* (2007) were the first to show that the*CEBPE* gene at 14q11 was a partner of the *IGH*gene in a case of B-ALL with t(14;14)(q11;q32). More recently, Pierini *et al.* (2011) demonstrated that chromosomal duplication and cryptic insertion produced a *CEBPE/IGH*fusion gene in B-cell ALL and that more than one *CEBPE/IGH*recombination can occur in a leukemic cell.

The *PAX5* gene, located on chromosome 9p13, encodes a transcription factor known as B-cell-specific activator protein (Familiades *et al.*, 2009). *PAX5* is one of nine human *PAX* genes (*PAX1-PAX9*) (Strachan and Read, 1994; Blake and Ziman, 2014). In view of its crucial role in normal B-lymphopoiesis, alteration in the *PAX5* gene is presumed to contribute to the leukemogenesis of B-ALL (Nebral*et al.*, 2009).

The *CDKN2A* gene, known as p16 (encoded protein), is a tumour suppressor gene located on chromosome 9p21 (http://www.omim.org/entry/600160). Deletion of the*CDKN2A* gene is a poor prognostic factor in adult but not in childhood B-ALL. This gene may play an important role in leukemogenesis in T-ALL and precursor B-ALL since monoallelic and biallelic deletions of this gene have been reported in both T-ALL and B-ALL (Van Zutven*et al.*, 2005; Kim*et al.*, 2009).

Deletion of *PAX5* and *CDKN2A* was reported by Kim *et al.* (2011); their comprehensive studies using FISH, G-banding and immunohistochemistry (IHC) showed that *PAX5* deletion was common in childhood and adult BALL. To our knowledge, the t(14;14) has been reported in only six cases of B-ALL (Berger *et al.*, 2001; Han *et al.*, 2008). Here, we report for the first time, the simultaneous involvement of an *IGH (*14q32*)/CEBPE* (14q11) fusion gene and a*PAX5/CDK2NA* concurrent deletion (9p13p21) in a complex translocation t(9;14;14) in a case of childhood B-ALL.

## Material and Methods

### Case report

A 5 year-old girl was admitted with a six-month history of anorexia and asthenia. Physical examination was remarkable for muco-cutaneous pallor and a weight of 17.5 kg. The patient presented with chest pain and 40 °C fever. She had no history of genetic diseases or known exposure to mutagenic agents. Complete blood analysis revealed a leukocyte count of 91.8 × 10^9^/L with 88% blast cells, a platelet count of 247 × 10^9^/L and hemoglobin of 8.6 g/dL. A bone marrow aspirate showed large leukemic cells with 90% blasts. Immunophenotyping was positive for CD10 (98%), CD19 (99%), CD22 (98%), CD33 (98%), CD34 (99%), CD38 (98%), CD45 (100%), CD79b (86%) and HLA-DR (98%), and negative for CD1a, CD2, CD4, CD5, CD7, CD11c, CD13, CD15 and CD56. The final diagnosis was B-ALL.

The first chemotherapy protocol (FRALLE 93) was started. After induction and consolidation, the complete first remission (2% blast cells) was achieved 2.5 years after admission. Five months later, she relapsed with 92% blast cells. A second chemotherapy protocol was started (COPRALL 2001), but two months later the patient presented a nosocomial infection. Following a third protocol (VANDA), 1.5 months later, a second complete remission was obtained with no blast cells detected. As no compatible family member was found, bone marrow transplant was not considered as an option for treatment. One year later, the blood analysis showed an infection with *Staphylococcus* and*Clostridium difficile* with 91% of blast cells. A fourth chemotherapy protocol was started, but unfortunately six months later the patient passed away.

### Chromosomal analysis

Chromosomal analysis of a bone marrow sample was done using a direct technique (Shiloh and Cohen, 1978). This method was based on short (25 min) incubation, immediately following aspiration, in a solution containing hypotonic KCl and colcemid that omitted the use of tissue culture medium. A conventional G-banding method was used for karyotyping. Clonal karyotype anomalies were described according to ISCN (Shaffer *et al.*, 2013).

### Fluorescence *in situ* hybridization

FISH was used to investigate whether t(14;14)(q11; q32) involved rearrangement of the genes *IGH* and *CEBPE* and was done as previously described by Akasaka *et al.* (2007). DNA was extracted from a BAC clone using a QIAGEN plasmid midi kit (Qiagen, Hilden, Germany) following the manufacturer's protocol. BAC DNA was labeled by nick translation (Roche Diagnostics, Mannheim, Germany) using a nick translation test kit (Abbott/Vysis, USA). Pretreatment of the probe and hybridization were done as previously described (Li *et al.*, 2004).

In order to map the chromosomal breakpoints, BAC clones were selected using the Human Genome Browser Gateway (version GRCh37/hg19). First, we used a dual-color break-apart *IGH* BAC clone, 442F20 and DJ998D24 (Jiang *et al.*, 2002) to detect rearrangement in the *IGH* gene (14q32). The centromeric 3' region of *IGH* was labeled with SpectrumOrange (442F20) and the telomeric 5' portion with SpectrumGreen (DJ998D24). One BAC clone (RP11-147E17), spanning the *CEBPE* locus at 14q11 and labeled with SpectrumGreen, was purchased from Invitrogen (Carlsbad, CA). The BAC clones spanning the *PAX5* gene (RP11-243F8, RP11-297B17 and RP11-344B23) were obtained from the Welcome Trust Sanger Institute (http://www.sanger.ac.uk). The centromeric 3' region of*PAX5* was labeled with SpectrumOrange (RP11-243F8 and RP11-297B17) and the telomeric 5' portion with SpectrumGreen (RP11-344B23).

We used a break-apart LSI *CDKN2A* BAC clone (RP11-149I2/70L8) (Welcome Trust Sanger Institute, http://www.sanger.ac.uk) to detect the deletion of *CDKN2A* gene (P16) on 9p21. We also used a *CEP9* probe (Abbott/Vysis, USA) to detect the deletion of chromosome 9p and a LSI *MYB* probe for chromosome 6 as an internal control.

The FISH signal was amplified and detected by using a conventional system that included a first layer of FITC-Avidin, a second layer of biotinylated-anti-Avidin and a third layer of FITC-Avidin (Cambio, Cambridge, UK). The BAC probe was initially hybridized to normal metaphases to confirm its location (data not shown). The FISH signal was captured using a Leica DMRXA fluorescence microscope (Leica, Wetzlar, Germany) and Q-FISH imaging software (Metasystems, Altlussheim, Germany) was used to scan and capture the images. At least 20 metaphases and/or 100 interphase nuclei were analyzed for each test. Each metaphase was counterstained with 4'-6-diamidino-2-phenylindole (DAPI) (Roche Diagnostics, Laval, QC, Canada).

## Results

### Chromosomal analysis

**Figure 1:**
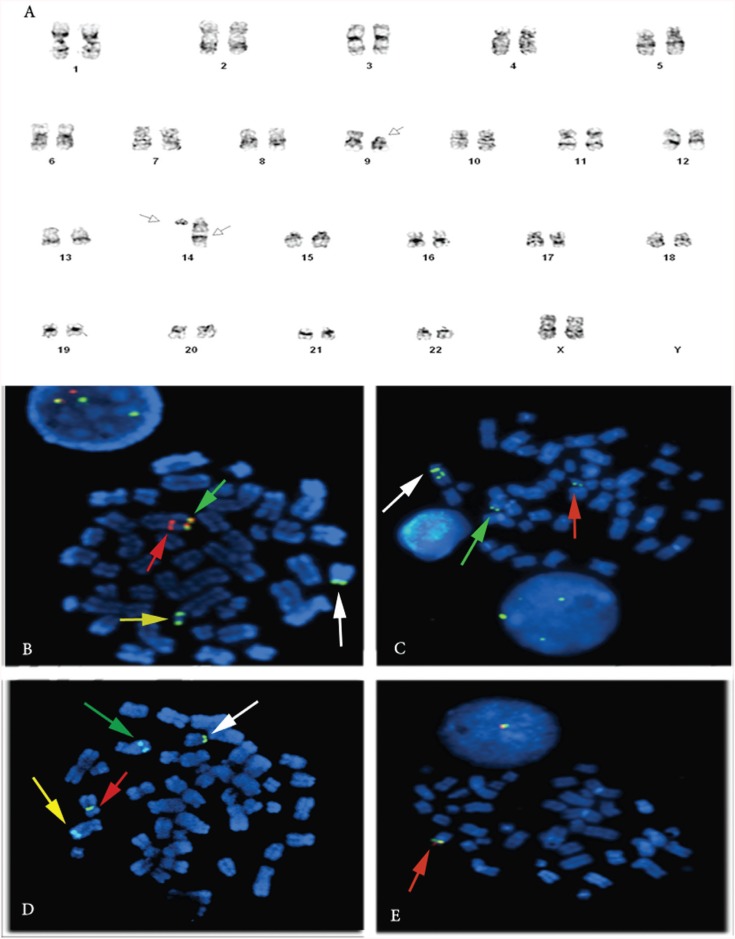
A. GTG-banded karyotypes of the probant bone marrow. A 46,XX,del(9)(p21),t(14;14)(q11;q32) karyotype was revealed at the onset of the disease. White arrows indicate abnormal chromosomes 9 and 14.**B.** FISH analysis of metaphase and interphase nuclei using a dual-color break-apart *IGH* probe, showing a normal fusion signal [orange (442F20)/green (DJ998D24); green arrow] on the terminal portion, a red (442F20) signal in the middle portion of the long arm of the larger der(14) chromosome (14q+) (red arrow), and a green (DJ998D24) signal on the smaller der(14) chromosome (14q-) (yellow arrow) and on deleted chromosome 9 (white arrow). **C.** FISH analysis of metaphase and interphase nuclei using a BAC (RP11-147E17)*CEBPE* probe, showing a large der(14) chromosome (14q+) with two green signals (RP11-147E17) (white arrow), a small der(14) chromosome (14q-) with only one green signal (RP11-147E17) (green arrow) and der(9) with a single green signal (RP11-147E17) (red arrow). **D.** FISH analysis of metaphase nuclei using the*CEP9* probe for the two chromosomes 9, showing two green signals on the centromeres, one on normal chromosome 9 (red arrow) and the other on deleted chromosome 9 (9p-) (white arrow). An*MYB* SpectrumAqua probe was used on both normal chromosomes 6 as an internal control and yielded two aquablue signals (green and yellow arrows). **E.** FISH analysis of metaphase and interphase nuclei using a dual-color break-apart*PAX5* probe, showing only one orange/green signal (RP11-243F8, RP11-297B17, RP11-344B23) on a normal chromosome 9 (red arrow).

A total of 20 metaphases were analyzed and only one karyotype was obtained, namely, 46, XX, del(9)(p21), t(14;14)(q11;q32) (Figure 1A).

### Fluorescence *in situ* hybridization

**Figure 2:**
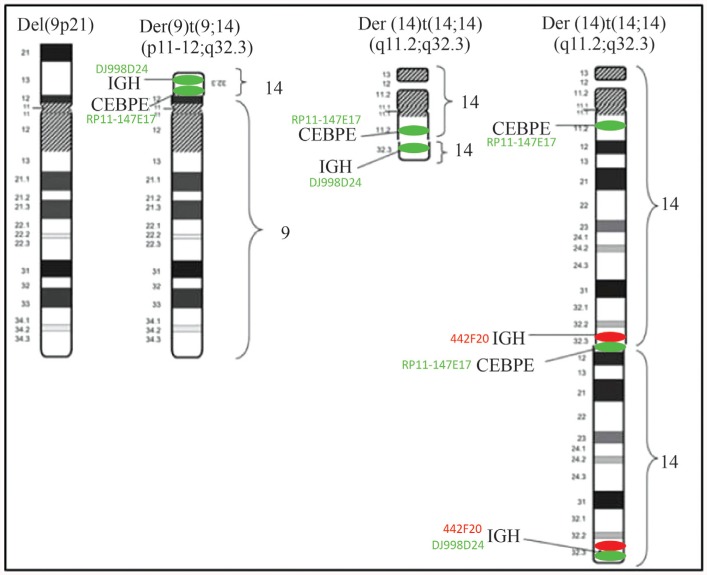
Ideograms of abnormal chromosomes 9 and 14 involved in the t(9;14;14), with the localization of the *IGH* and*CEBPE* genes labeled by three BACs – 442F20 (red), J998D24 (green) and RP11-147E17 (green) - used as probes in FISH experiments.

In each analyzed cell, we observed two abnormal derivative chromosomes 14 (Figures 1B and 2). Two FISH signals were observed on the large derivative chromosome 14: an orange signal at the translocation breakpoint 14q32 (3' part of the *IGH* break-apart probe, 442F20) and an orange/green fusion signal at the normal *IGH* locus (442F20 and DJ998D24). One green signal corresponded to the non-rearranged 14q11 locus and the second green signal (5' part of the *CEBPE* gene) to the rearranged*IGH* locus (14q32). The former locus was translocated from the small derivative 14 (3' part of the *CEBPE* gene). The small derivative chromosome 14 showed a single green signal at the translocation breakpoint 14q11 (5' part of the *IGH* breakpoint probe DJ998D24). No normal cells were seen in this analysis.

To detect rearrangement of the *CEBPE* gene on the 14q11 locus, we used a FITC-labeled BAC green probe (RP11-147E17 and RP11-68M15) (http://www.sanger.ac.uk). Figures 1C and 2 show that two green FISH signals were detected on the large derivative chromosome 14; a single green signal (3' part of the *CEBPE* BAC probe) was also detect at the translocation breakpoint (14q11) on the small derivative chromosome 14. Another green signal was observed on the derivative chromosome 9 (9p21) on the 3' part of the *CEBPE* BAC probe. To investigate the breakpoint on chromosome 9, we used a dual-color breakapart *PAX5* BAC probe (RP11-243F8, RP11-297B17 and RP11-344B23) (http://www.sanger.ac.uk). Only one orange/green signal was seen on a normal chromosome 9, indicating that the *PAX5* gene on the other chromosome 9 was deleted.

Figures 1D and 2 show the metaphase FISH analysis using the*CEP9* probe for the two chromosomes 9,with two green signals on the centromeres: one on normal chromosome 9 and the other on derivative chromosome 9 (9p-). An *MYB* SpectrumAqua probe was used on both normal chromosomes 6 as an internal control and showed two aquablue signals.Figures 1E and 2 show that metaphase FISH analysis using the*CEP9* probe (9p11-q11) confirmed deletion of the*PAX5* locus. Interphase nuclei FISH analysis using break-apart *CDKN2A* probe for the two chromosomes 9 yielded two green signals for *CEP9* and only one red signal for*CDKN2A* at 9p21 (data not shown).

Based on the GTG banding and FISH results, the most likely interpretation of the karyotype is a cryptic complex translocation involving chromosomes 14 and 9 short arm. The derivative chromosome 9 was positive with BAC probes targeting the IGH and *CEBPE* loci, and negative with BAC probes targeting the *PAX5* locus. This led to the following interpretation of the FISH karyotype: 46,XX,del(9)(p21),t(14;14)(q11;q32).isht(14;14;9) (q11.2; q32.3;p21) (RP11-147E17+, DJ998D24+; RP11-147E17+, 442F20+; RP11-147E17+, DJ998D24+, RP11-243F8-, RP11-297B17-, RP11-344B23-).

## Discussion

Based on GTG banding alone the interpretation of the karyotype was 46,XX,del(9)(p21), t(14;14)(q11;q32). However, in our case, the FISH results using the BAC locus probe specific for *CEBPE* and the break-apart probe specific for*IGH* (442F20 and DJ998D24) showed signals corresponding to the 3' of *CEBPE* and 5' of *IGH* on deleted chromosome 9, suggesting the presence of an *IGH-CEBPE* fusion gene. Based on the FISH results, the most probable interpretation of this karyotype was a complex translocation t(9;14;14) associated with a large deletion within 9p and a duplication involving at least the fusion gene *IGH-CEBPE*.

Chromosome *in situ* hybridization with BAC specific for the*CEBPE* and *IGH* genes revealed a hybridization profile compatible with rearrangement of the *CEBPE* (14q11.2) and*IGH* (14q32) loci. This finding suggested the presence of*IGH-CEBPE* fusion on the small derivative chromosome 14 and*CEBPE-IGH* fusion on the derivative large chromosome 14, a conclusion in agreement with Han *et al.* (2008), who demonstrated the involvement of*IGH* and *CEBPE* genes in t(14;14)(q11;q32) in B-ALL.

Intra-chromosomal translocations involving *IGH* and*CEBPE* have been described in childhood ALL and result in the upregulation of *CEBPE* expression, suggesting that*CEBPE* plays a possible role in the development of B-ALL (Akasaka *et al.*, 2007). The presence of an *IGH-CEBPE* fusion on 9p suggests that a duplication and large deletion occurred simultaneously with a translocation involving 9p12, 14q11 and 14q32. This complex single rearrangement event led to the formation of an*IGH-CEBPE* fusion gene and concurrent deletion of*PAX5* and *CDKN2A* on 9p. Simultaneous deletion of *PAX5* and *CDKN2A* is a common event in leukemogenesis and most ALL patients with a deletion of *PAX5* have a concurrent deletion of *CDKN2A* (Kim*et al.*, 2009).

Although we cannot exclude that the translocation t(14;14) and deletion 9p are two independent events, we believe that the presence of a second set of*IGH-CEBPE* fusion genes at the breakpoint of 9p reflects the activity of a DNA repair mechanism such as non-homologous end joining (NHEJ). This pathway repairs double-strand breaks with no homologous sequence and usually underlies deletions and duplications at the breakpoints of the two broken DNA ends to be tied (Zhang *et al.*, 2009a,b). If NHEJ is involved, then only one event was needed to produce a double set of *IGH-CEBPE*and the concurrent deletion of *PAX5* and *CDKN2A*. The occurrence of all these aberrations probably potentiated the aggressive refractory leukemia in our patient. Our case increases the number of B-ALL patients with t(14;14) in the literature to seven. Table 1 summarizes the clinical, hematological, immunophenotypic and genetic findings of these patients.

## Conclusion

Our B-ALL finding revealed a complex translocation t(9;14;14)(p12;q11;q32) accompanied by the formation of an *IGH-CEBPE* fusion gene and its duplication, and the concurrent deletion of *PAX5* and*CDKN2A* on 9p. To our knowledge, this is the first report to identify four important steps of leukemogenesis simultaneously in one event.
